# Spontaneous Regression of Intrahepatic Adenocarcinoma after Needle Biopsy: A Case Report and Literature Review

**DOI:** 10.70352/scrj.cr.25-0613

**Published:** 2026-01-30

**Authors:** Koetsu Inoue, Tatsunori Bandai, Naota Okabe, Masahiro Hiruta, Hisashi Oshiro, Yuki Mizusawa, Hidetoshi Aizawa, Yuhei Endo, Fumiaki Watanabe, Hiroshi Noda, Toshiki Rikiyama

**Affiliations:** 1 Department of Surgery, Saitama Medical Center, Jichi Medical University, Saitama, Saitama, Japan; 2 Department of Surgery, Tohoku University Graduate School of Medicine, Sendai, Miyagi, Japan; 3 Department of Pathology, Saitama Medical Center, Jichi Medical University, Saitama, Saitama, Japan

**Keywords:** intrahepatic cholangiocarcinoma, spontaneous tumor regression, CD8-positive T cell

## Abstract

**INTRODUCTION:**

Spontaneous tumor regression (STR) is a rare phenomenon in which cancer cells partially or completely disappear without treatment. We report a case of intrahepatic cholangiocarcinoma demonstrating STR following endoscopic ultrasonography-guided fine-needle aspiration (EUS-FNA).

**CASE PRESENTATION:**

A 77-year-old male presented with acute cholecystitis 1 day after percutaneous coronary intervention for acute myocardial infarction. Conservative treatment and endoscopic retrograde gallbladder drainage were performed due to antiplatelet therapy. Cytology of bile juice unexpectedly revealed adenocarcinoma. Further imaging, including CT and mapping biopsy, failed to detect a tumor. Laparoscopic cholecystectomy with gallbladder bed resection showed no histological evidence of malignancy. Four months later, surveillance CT revealed a 15 × 15 mm lesion in segment 3 of the liver. EUS-FNA confirmed adenocarcinoma. Laparoscopic left lateral resection was performed; however, no viable cancer cells were identified, and the lesion was replaced by epithelioid granulomas. Immunohistochemistry demonstrated dense infiltration of CD8-positive cytotoxic T cells, suggesting an immune-mediated regression of the tumor.

**CONCLUSIONS:**

This case highlights the possibility of tumor regression in intrahepatic adenocarcinoma following EUS-FNA, potentially triggered by an immune response.

## INTRODUCTION

Spontaneous tumor regression (STR) is a rare phenomenon characterized by partial or complete disappearance of a tumor without treatment known to cause regression.^1,2)^ It was first defined by Dr. Warren H. Cole and Dr. Tilden C. Everson in 1956.^3)^ STR can occur throughout the body, including the gastrointestinal tract, and its frequency varies by cancer type.^4)^ Previous studies estimated the incidence of STR as 60000–100000 cases,^5,6)^ with renal cell carcinoma, melanoma, and neuroblastoma being most frequently reported.^7)^ STR in gastrointestinal cancers has been observed more than twice as often in males compared with females.^7)^

Proposed mechanisms for STR include immunological responses, inflammation, apoptosis, epigenetic modification, tumor inhibition by growth factors or cytokines, hormonal mediation, and ischemia.^8,9)^ Among these, immunological response is the most widely recognized hypothesis. Because T cell-mediated immune activity induces durable effects with immunotherapy, spontaneously activated T cell-mediated immunity may be a key driver of STR.^10)^ Although STR occurs in the absence of conventional therapies, such as chemotherapy and radiotherapy, elucidating its mechanism could guide the development of novel cancer treatments.

Cholangiocarcinoma represents approximately 10% of hepatobiliary tumors and is considered rare.^11)^ To date, only two cases of cholangiocarcinoma associated with STR have been reported.^12,13)^ We describe a rare case of intrahepatic cholangiocarcinoma that underwent STR following endoscopic ultrasonography-guided fine-needle aspiration (EUS-FNA) and emphasize the role of a naïve antitumor immune response in this regression.

## CASE PRESENTATION

**Fig. 1:**
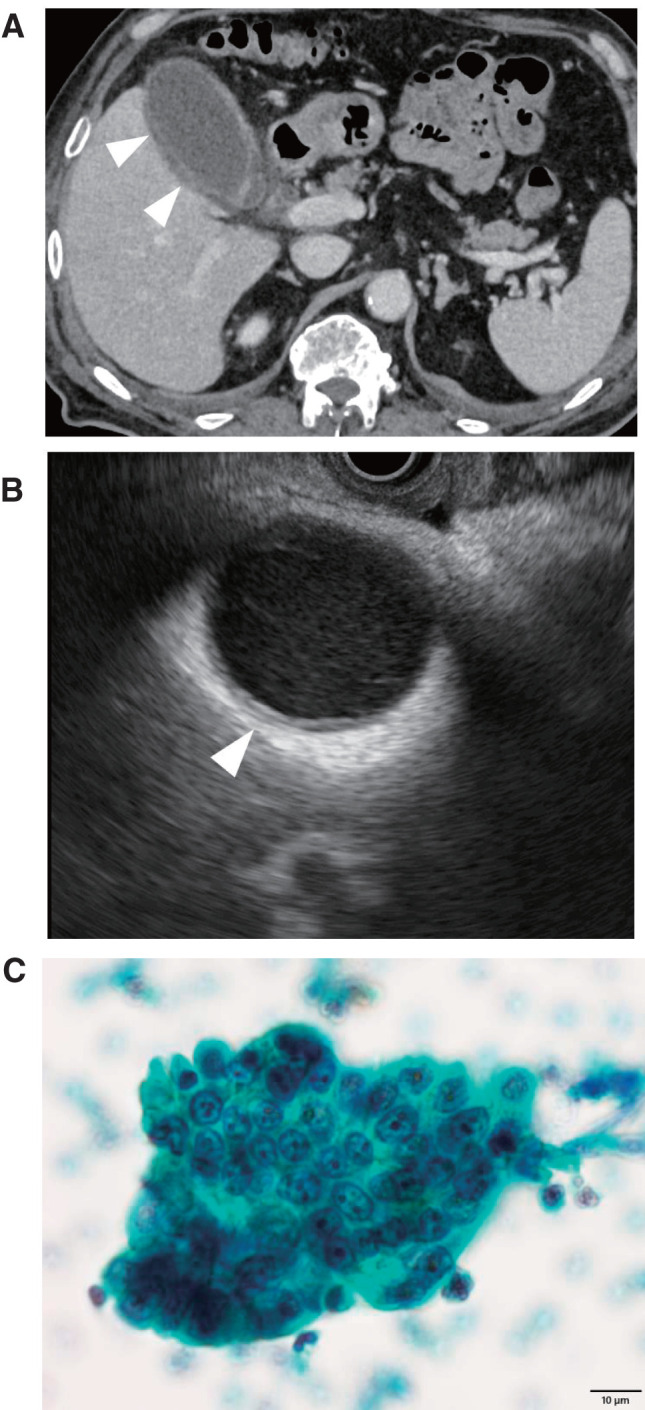
Imaging and cytology at initial admission. (**A**) Axial CT showing gallbladder wall thickening (white arrowheads). No bile duct dilation or tumor was observed. (**B**) EUS demonstrating gallbladder wall thickening (white arrowheads), consistent with CT findings. No tumor was detected. (**C**) Cytology of bile juice showing adenocarcinoma. Scale bar: 10 μm. EUS, endoscopic ultrasonography

**Fig. 2:**
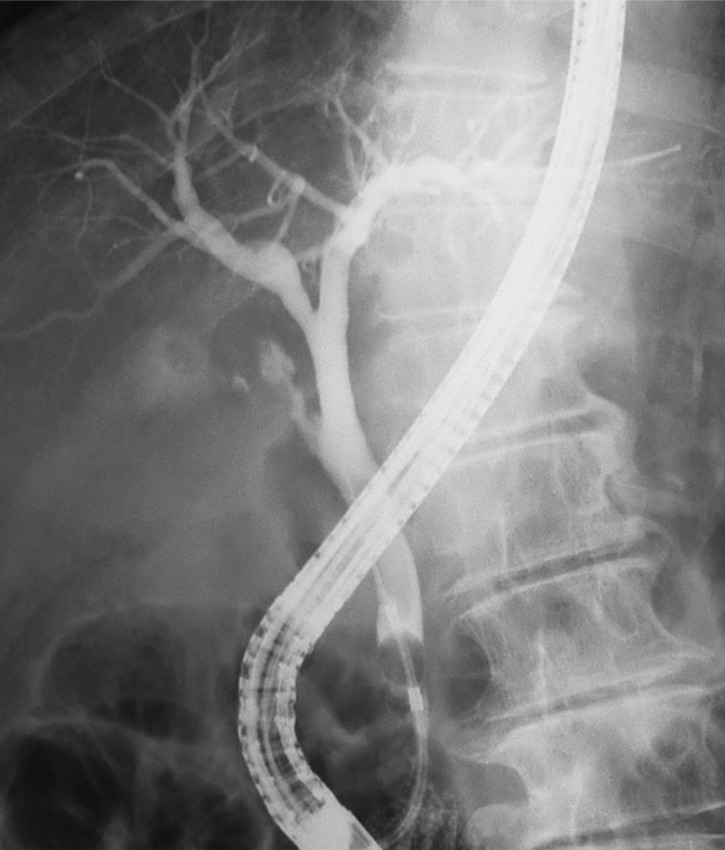
ERCP showing a smooth bile duct without dilation. ERCP, endoscopic retrograde cholangiopancreatography

**Fig. 3:**
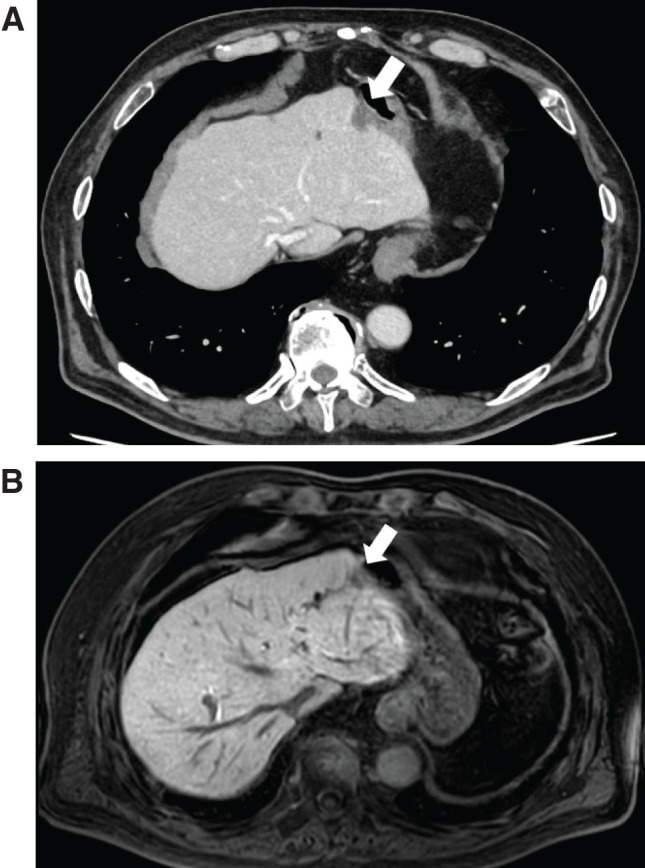
Imaging 4 months after laparoscopic cholecystectomy with gallbladder bed resection. (**A**) Surveillance axial CT showing a tumor in segment 3 (white arrow). No bile duct dilation was observed. (**B**) Axial T1-weighted MRI showing a tumor in segment 3 (white arrowhead).

**Fig. 4:**
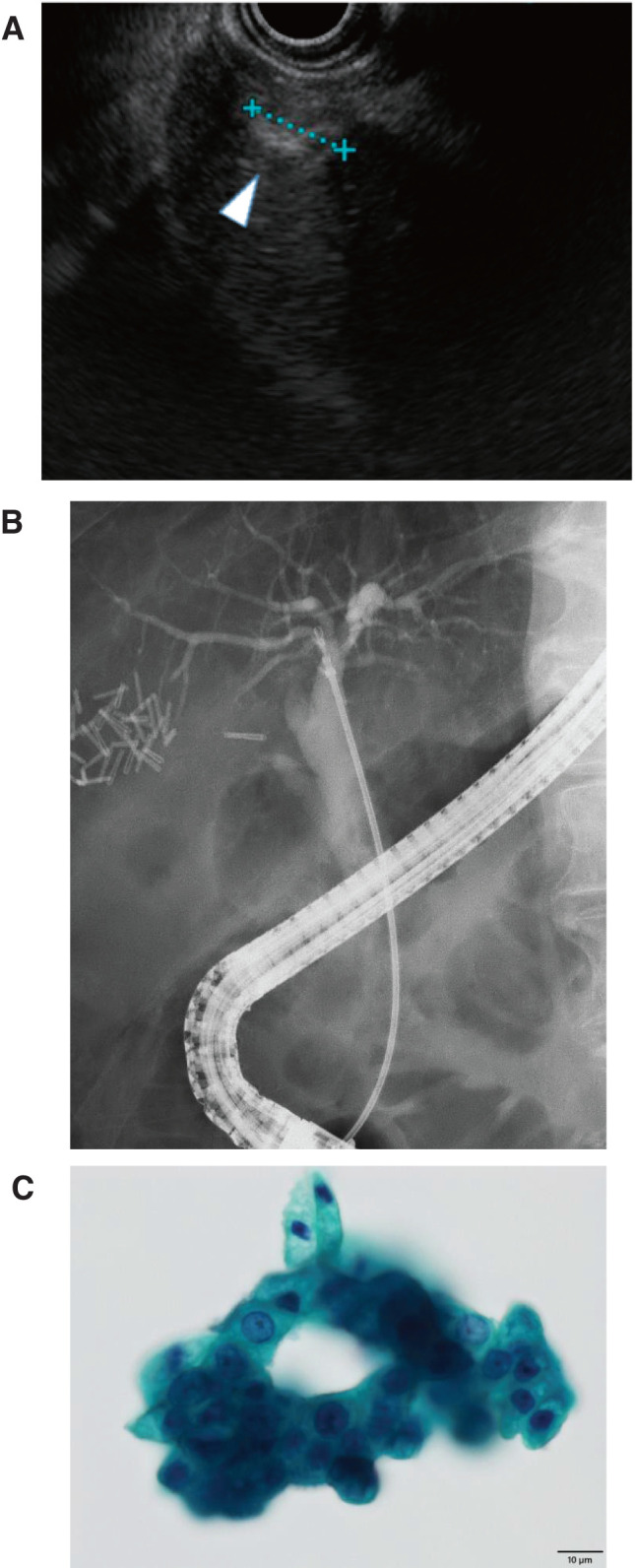
Imaging and cytology before laparoscopic left lateral resection. (**A**) EUS demonstrating a tumor in segment 3 (white arrowhead), consistent with CT findings. (**B**) ERCP showing a smooth bile duct without dilation. (**C**) EUS-FNA sample from the tumor showing adenocarcinoma. Scale bar: 10 μm. EUS, endoscopic ultrasonography; EUS-FNA, endoscopic ultrasonography-guided fine needle aspiration

**Fig. 5:**
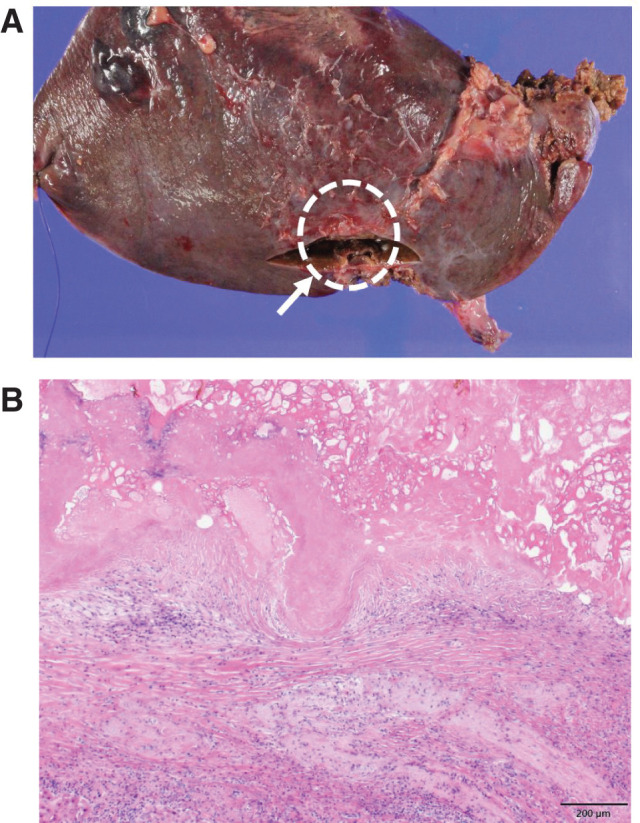
Surgical specimen and postoperative histopathology. (**A**) Surgical specimen from left lateral segmentectomy showing a lesion in segment 3 (white arrow). (**B**) Postoperative histopathology showing epithelioid granuloma replacing the lesion without viable cancer cells. Scale bar: 200 μm.

**Fig. 6:**
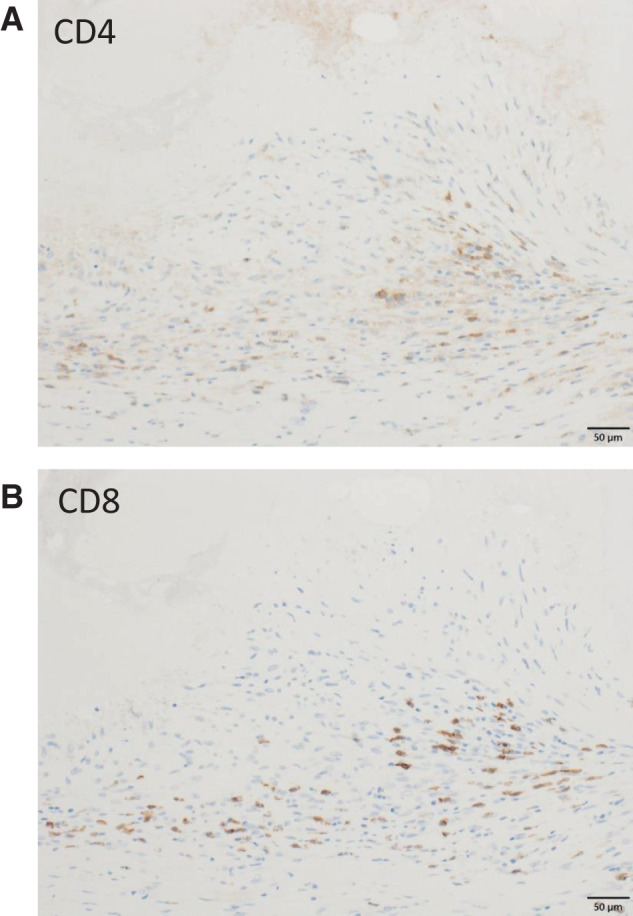
Immunohistochemical staining for CD4 and CD8. (**A**) CD4, Scale bar: 50 μm (**B**) CD8, Scale bar: 50 μm.

A 77-year-old male patient was admitted to our department for cholecystitis 1 day after undergoing percutaneous coronary intervention for acute myocardial infarction. He presented with fever and right upper abdominal pain. CT revealed gallbladder wall thickening without stones. Due to his antiplatelet therapy, conservative management and endoscopic retrograde gallbladder drainage were performed. Cytology of bile juice unexpectedly revealed adenocarcinoma. Subsequent CT and endoscopic ultrasonography (EUS) demonstrated gallbladder wall thickening without a discrete tumor (**Fig. 1**). After resolution of cholecystitis, endoscopic retrograde cholangiopancreatography (ERCP) showed a smooth bile duct with no evidence of extra-bile duct cancer (**Fig. 2**). Mapping biopsies were negative for malignancy, and tumor markers including carcinoembryonic antigen (CEA) and carbohydrate antigen 19-9 (CA19-9) were not elevated. Inflammatory markers, including C-reactive protein, normalized after resolution of cholecystitis and showed no persistent elevation during subsequent follow-up. These findings raised suspicion of gallbladder cancer as the cause of cholecystitis. Laparoscopic cholecystectomy with gallbladder bed resection was performed, but histology revealed no malignancy. Four months later, surveillance CT and MRI identified a 15 × 15 mm tumor in segment 3 (**Fig. 3**). No bile duct dilation was noted. EUS confirmed a tumor (11 mm in diameter), which was diagnosed as adenocarcinoma by FNA (**Fig. 4**). Because adenocarcinoma had been detected in bile juice, bile duct invasion was suspected. However, repeat ERCP showed no obstruction, and mapping biopsies remained negative. Given the cytological diagnosis of adenocarcinoma, laparoscopic left lateral sectionectomy was performed approximately 6 weeks after EUS-FNA. Histopathology demonstrated absence of viable cancer cells, with necrotic tissue replaced by epithelioid granulomas (**Fig. 5**). Histological examination of the non-lesional liver parenchyma revealed no advanced fibrosis or significant chronic inflammatory changes. These results suggest that STR had occurred following EUS-FNA. As immunological response is a recognized mechanism of STR, immunohistochemistry was performed, revealing cytotoxic T cell (CD8-positive T cell) infiltration into the lesion, supporting immune-mediated tumor regression (**Fig. 6**). No postoperative adjuvant chemotherapy was administered. Given the absence of viable tumor cells in the resected specimen and the interpretation of spontaneous regression, the patient has been managed with careful surveillance alone.

## DISCUSSION

STR is an uncommon event described in the medical literature, and most reports are single cases due to its rarity. Minacapelli et al. reviewed 390 cases of STR in gastrointestinal malignancies, with hepatocellular carcinoma (193 cases, 49.5%) and colorectal cancer (52 cases, 13.3%) being most frequent.^7)^ Only two cases of cholangiocarcinoma (0.51%) were documented. In one case, tumor regression occurred after right portal vein ligation, and histopathology showed no residual cancer cells.^13)^ In the other, tumor shrinkage was observed without treatment; however, regrowth occurred, necessitating liver resection, and no potential trigger was identified.^12)^ Consequently, the present case represents the first report of intrahepatic cholangiocarcinoma showing STR following EUS-FNA.

Because data on STR remain limited, its mechanism is not well understood. Proposed explanations include immunological response, inflammation, apoptosis, epigenetic modification, tumor inhibition by growth factors or cytokines, hormonal mediation, ischemia,^3)^ trauma, or infection.^2,14)^ In this case, EUS-FNA may have induced trauma, contributing to STR. Surgical stimulation has also been reported to activate local immune responses.^15)^ Moreover, in a review of 176 cases of STR, 40% were related to operative trauma.^2)^ EUS-FNA may enhance the release of tumor-associated antigens, promote dendritic cell activation, and facilitate cross-presentation to cytotoxic CD8-positive T cells.^15)^ Immunohistochemical staining revealed cytotoxic T cell infiltration around epithelioid granulomas, suggesting that EUS-FNA possibly triggered an immune-mediated regression, consistent with previously described mechanisms.

Immunotherapy is now a standard treatment for several malignancies, often used as a first-line therapy, and complete response (CR) is not uncommon in specific cancer types.^16)^ Histopathological assessment of lesions after CR could yield critical insights into the hallmarks of antitumor immunity. However, opportunities to analyze tissue showing CR after immunotherapy are rare, and only a few cases have been reported.^17–19)^ Immunohistochemical analysis demonstrated infiltration of CD8-positive T cells within and around the epithelioid granulomas. However, infiltration of CD8-positive T cells alone does not establish tumor antigen specificity, nor does it prove a causal role in tumor regression. Granuloma formation can occur in a variety of inflammatory or immune-mediated conditions and should therefore be interpreted with caution. Nevertheless, previous reports of immune checkpoint inhibitor-associated complete responses and sarcoid-like reactions have described granulomatous lesions accompanied by cytotoxic T cell infiltration in the context of effective antitumor immunity.^20)^ In this regard, the granulomatous reaction observed in the present case may represent a histopathological pattern *compatible with*, but not specific for, immune-mediated tumor regression. We therefore interpret these findings as supportive but not definitive evidence of an immune response potentially involved in the spontaneous regression observed in this patient. Additional factors such as infection, enterobacterial flora, and genetic background may also influence STR mechanisms.^18)^

The absence of malignancy in the gallbladder strongly suggests that the positive bile juice cytology originated from the intrahepatic lesion rather than from gallbladder cancer. Therefore, the initial episode was most consistent with acalculous cholecystitis following acute myocardial infarction, rather than malignancy-related cholecystitis. Regarding the segment 3 lesion, it is possible the tumor was already present at the time of the initial presentation but was below the detection threshold of CT and EUS, since microscopic or early-stage intrahepatic cholangiocarcinoma can escape radiological detection, particularly in the setting of acute inflammation. This possibility does not contradict our interpretation but rather supports the notion that tumor regression occurred after EUS-FNA, when the lesion had become radiologically detectable and subsequently regressed. In addition, the interval between EUS-FNA and laparoscopic left lateral sectionectomy was approximately 6 weeks. Previous studies have reported that spontaneous regression of gastrointestinal cancers typically occurs over a period of several weeks to several months.^7)^ Therefore, the observed interval in the present case is consistent with previously reported time courses and supports the biological plausibility of immune-mediated tumor elimination.

A potential limitation of this case is the possibility of initial misdiagnosis. Immunohistochemical staining for biliary epithelial markers such as CK7, CK19, CK20, and MUC1 was not performed on the EUS-FNA specimen, primarily due to limited material availability at the time of diagnosis. Nevertheless, we believe the diagnosis of adenocarcinoma is reliable for several reasons. First, both bile juice cytology and EUS-FNA independently demonstrated malignant epithelial cells with adenocarcinomatous features. Second, both diagnostic modalities are known to have very high specificity, approaching 100% in several reports, even though sensitivity may be limited. Previous studies have reported a specificity of up to 100% and an accuracy of approximately 88.2%.^21,22)^ Similarly, EUS-FNA has shown specificity of 100% and accuracy of 79%.^23)^ Third, the cytological morphology was inconsistent with reactive or benign biliary epithelium. Regarding alternative diagnoses, inflammatory pseudotumor and IgG4-related disease should be considered. These entities were considered unlikely because (i) malignant cells were identified cytologically, (ii) no storiform fibrosis or obliterative phlebitis was observed histologically, and (iii) the clinical course and imaging findings were inconsistent with inflammatory pseudotumor. Therefore, the diagnosis of adenocarcinoma in this case is highly reliable, and STR is the most plausible explanation.

## CONCLUSIONS

This case demonstrates STR of intrahepatic cholangiocarcinoma following EUS-FNA. Understanding the mechanisms underlying STR may inform development of novel cancer therapies, warranting further investigation.

## DECLARATIONS

### Availability of data and materials

All the data are included within the article.
